# Orthodontic Treatment of Dogs during the Developmental Stage: Repositioning of Mandibular Canine Teeth with Intercurrent Mandibular Distoclusion

**DOI:** 10.3390/vetsci9080392

**Published:** 2022-07-29

**Authors:** Małgorzata Peruga, Grzegorz Piątkowski, Jakub Kotowicz, Joanna Lis

**Affiliations:** 1 Individual Medical Practice, 93-410 Łódź, Poland; 2 GREG-Dent Dental Labor, 91-163 Łódź, Poland; g.j.piatkowski@gmail.com; 3 Individual Veterinary Practice, 37-700 Przemyśl, Poland; jakub.kotowicz@gmail.com; 4 Department of Maxillofacial Orthopaedics and Orthodontics, Wroclaw Medical University, 50-376 Wrocław, Poland; stomjan@gmail.com

**Keywords:** dog, orthodontic treatment, mandibular distoclusion, canine

## Abstract

**Simple Summary:**

Orthodontic treatment of dogs in the growth spurt is most effective. It gives a permanent shift of the teeth and a change in occlusion. Treatment does not require retention. Early diagnosis and proper treatment will protect dog in the early stage of life from tooth removal. The use of acrylic appliances is a sure method of treating tooth displacement defects with the accompanying displacement of the mandible, which is a daily practice in these tests.

**Abstract:**

Linguoverted mandibular canines are relatively rare among craniofacial abnormalities, and they are an isolated anomaly. They are most often caused by non-genetic factors such as persistent deciduous canine teeth or trauma coinciding with the eruption of permanent teeth. Another factor may be mandible narrowing or underdevelopment in the transverse dimension and vestibular inclination of the maxillary canine teeth. This article presents a procedure based on three cases where the position of the mandibular canine tooth was corrected using human orthodontic appliances modified to affect the canine dental system. The incline of the appliance was made to stimulate the protrusion of the mandible while the teeth were closing. After approximately 4 weeks, the lower canine teeth moved along the incline of the appliance, and tilt toward the flews was achieved.

## 1. Introduction

Orthodontics is a field of dentistry that deals with treating malocclusion, maxillofacial defects, and the correction of dental abnormalities. Appropriate redirection of orthodontic and physiological forces, such as pressure during jaw locking, can shift the tooth in the right direction in a controlled manner and the correct position of the mandible and the maxilla can be achieved. [1,2,3,4,5].

The fossil record of early mammals indicates a gradual reduction in the number of deciduous teeth associated with occlusion changes during an organism’s lifetime. The lateral teeth gradually migrate forward as the front molars wear down, making room for new molars [4,5,6]. Mammals differ from other vertebrates due to the fact that they have a limited ability to replace their teeth. Most mammals replace them only once in their lifetime. In response, many mammals have evolved to protrude the mandible or change the position of the temporomandibular joint and therefore change the occlusion. These features can protect against occlusion disorders, and they are of assistance during the treatment of occlusion abnormalities.

No significant dental or skeletal defects are usually found during the examination of fossil animal skulls. Archaeological research has allowed the determination of the appearance patterns of different species [6]. The food industry revolution significantly disrupted the development of the chewing apparatus. Therefore, one of the factors amplifying the occurrence of malocclusion are changes in food consistency. They influenced the evolution of the facial part of the human skull structure and led to the defects of the chewing apparatus which are more and more often observed in pets.

As an animal model, dogs are mainly used to study the etiopathogenesis of dental caries and the physiological wear of the teeth. It is, however, necessary to remember the differences between dog and human dentition. Dogs have 42 permanent teeth that are unevenly spaced over the dental arches. The maxillary dentition consists of 20 and the mandibular of 22 teeth. The maxillary fourth premolars and the mandibular first molars that are in contact when the jaws close are considered the largest. Humans have 32 teeth equally spaced in each quadrant, all of which are in contact during closing. Therefore, only molars can be compared between humans and dogs [7].

The experience gained in treating humans can and should be used to treat pets because they have—like all mammals—tecodontic teeth defined as teeth held in the alveolar process on the jaw bone and connected to it with a periodontal ligament. Orthodontic biomechanics is based on its reconstruction [6]. The development of the permanent tooth bud initiates the resorption of the deciduous tooth root and the reconstruction of the surrounding bone. The bud of the lower canine tooth cuts lingually in relation to the resorbed deciduous canine tooth. Then, after the deciduous tooth exfoliates, it changes to the vestibular direction. This process can be disturbed—similar to humans. The causes include injuries during tooth eruption and narrowing of the jaw. In miniature breeds the most common cause is the persistent deciduous mandibular canine. However, this disorder can also appear in large breeds.

One of the negative consequences of leaving an incorrectly erected mandibular canine is mandible narrowing. This is a direct cause of the inability to eat due to constant irritation of the palate mucosa, weight loss, and behavioral changes. The pressure on jaw locking in dogs is higher, so it can be safely concluded that the treatment process will be much faster.

The article aims to present orthodontic methods of distoclusion treatment with intercurrent dental abnormalities and damage to the hard palate in dogs. The treatment was performed with modified appliances used to treat similar defects in humans.

## 2. Materials and Methods

Three dogs—two male dogs and one female dog—were treated during puberty. The dogs that received treatment were purebred dogs. For the treatment, modified appliances were used, the same as those used for the treatment of malocclusion in humans, and they were used here as a result of close cooperation between the orthodontist and the veterinarian. In the treatment, the structure and the arrangement of the teeth of humans and dogs as well as the structure of joints were different, and it was preserved. In all cases, the intended effect was achieved, and it persists.

## 3. Case Reports

### 3.1. Patient 1—German Shepherd

A 7-month-old German Shepherd (male) was brought to the orthodontic clinic due to a lingually tilted fourth left mandibular tooth. The interview and the physical examination revealed the absence of comorbidities. The dog was from a regular litter, born on time and without complications.

**Figure 1:**
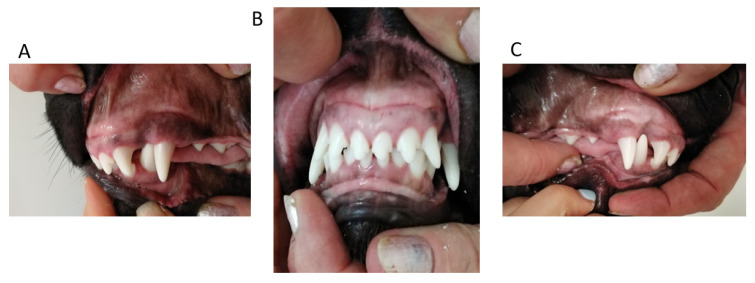
First case. Occlusion before treatment. (**A**) left side, (**B**) en face, (**C**) right side.

**Figure 2:**
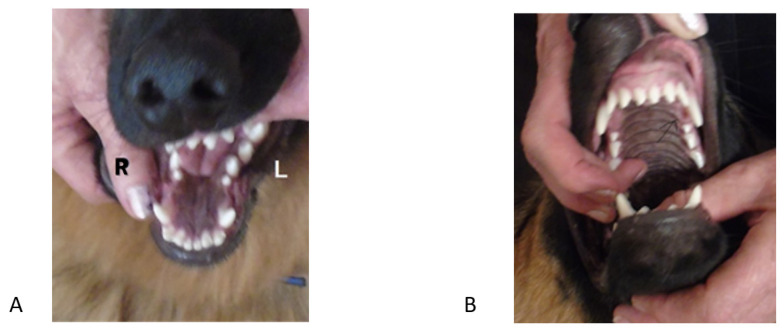
First case. Images in the occlusal projection. (**A**) mandibular arch, (**B**) maxillary arch—the arrow marks the recess. right (R) and left (L).

A 2 mm deep cavity was located on the hard palate, without any pathological changes, nor exudate from the cavity. The left mandibular canine was tilted toward the space behind the maxillary canine, causing the mandible to deviate to the right. Additionally, distoclusion was diagnosed (Figure 1 and Figure 2). Based on an interview with the owner, it was found that the dog was in pain, which was not associated with intaking food or water or playing with soft objects.

The animal’s general condition was found to be good—appropriate for the breed and age. After presenting the diagnosis and the treatment plan, the owner decided to start orthodontic treatment.

During the first visit, upper and lower dental arch impressions were taken with Kromopan (Lascod) alginate mass on upper disposable Duralock plastic trays (size M). A wax impression of the intercuspal position was also taken. Sedation was not necessary due to good cooperation with the dog and the owner. The impressions were disinfected with Durr Dental MD 520 for immersion disinfection in the clinic.

In the technical laboratory, the impressions were decontaminated with a disinfecting preparation intended for quick and very effective disinfection of alginate impressions (ZETA 7 SPRAY, Zhermack^®^). The impressions were cast from class IV synthetic gypsum, maintaining the appropriate proportions and manufacturer’s recommendations to obtain consistent linear dimensions of the finished gypsum models. The plaster models—after casting and setting—were trimmed, retaining the mapped tissues, teeth, and palate used for the task. Then, the models were joined using the wax impression.

During the next visit, a construction bite impression was made with wax, forcing the mandible to be properly positioned in relation to the maxilla and allowing orthodontic work to be performed: two inclined planes, separately on the left and right sides of both jaws.

The construction bite was submitted to the technical laboratory. On its basis, gypsum models were restrained with articulation plaster with appropriate expansion properties, which protected them against uncontrolled occlusion elevation.

Then, two separate acrylic inclined planes were made with different inclination angles, according to the design made by the veterinarian.

At the next clinical visit, the covers were cemented with Ketac Cem EASYMIX 30 g + 12 mL and a control visit was recommended in two weeks. After this time, the orthodontic appliance was removed from the tooth surface using orthodontic ring forceps. Cement residues from the teeth were removed with an applicator; the appropriate type of cement allowed the teeth to be cleaned without the need for anesthesia.

**Figure 3:**
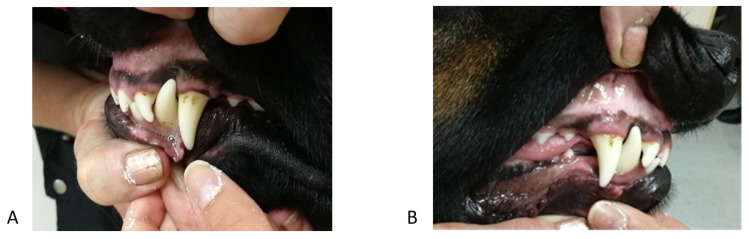
First case. Occlusion after treatment. (**A**) left side, (**B**) right side.

**Figure 4:**
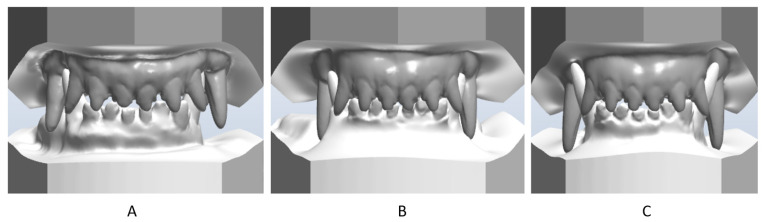
First case. Comparative en face occlusion scans—canine position and maxilla and mandible relationship. (**A**) before treatment, (**B**) after 2 months of treatment, (**C**) in the retention period (after 12 months of treatment).

The occlusion after 12 months of treatment is displayed in Figure 3 and Figure 4.

### 3.2. Patient 2—German Pointer

**Figure 5:**
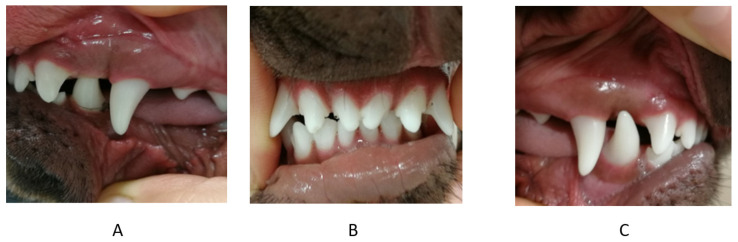
Second case. Intraoral photographs in full occlusion before treatment—press of the mandible against the maxilla, the recess behind the maxillary canine and the visible asymmetry of the arches with a disturbed plane. (**A**) left side, (**B**) en face, (**C**) right side.

**Figure 6:**
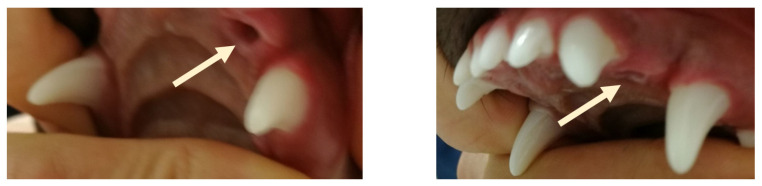
Second case. Intraoral photographs of the maxilla—a visible recess in the palate mucosa caused by the mandibular canine (arrow).

**Figure 7:**
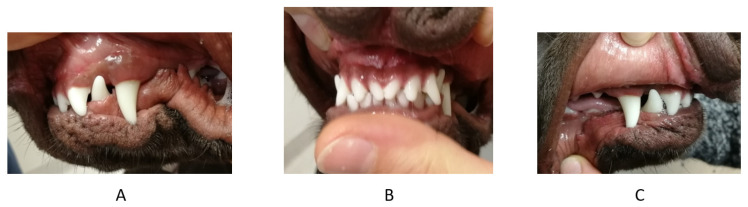
Second case. Intraoral photographs in full occlusion after treatment—press of the mandible against the maxilla. (**A**) left side, (**B**) en face, (**C**) right side.

The owner of a six-month-old female pointing dog reported to the clinic as he had noticed his dog’s teeth were incorrectly positioned. During the examination, the mandibular left fourth tooth was tilted. The owner did not report any other comorbidities. The dog was from a regular litter, born on time, without any complications. There was a noticeable asymmetry of the skull near the frontal bones, an intraoral recess in the palate, 5 mm deep, intense reddening, no exudate from the cavity was noticed. The mandibular canine was tilted into the space behind the maxillary canine, causing the mandible to deviate to the opposite side, with plane disturbance and a distoclusion. (Figure 5 and Figure 6). The owner decided to start orthodontic treatment. The same protocol as with the previous patient was followed. The patient and the owner were very cooperative, so there was no need for sedation during the impression taking or during appliance assembly and disassembly. Impressions and photographs were taken for a second time 2 months after the end of the treatment (Figure 7).

### 3.3. Patient 3—Short-Haired Weimaraner

**Figure 8:**
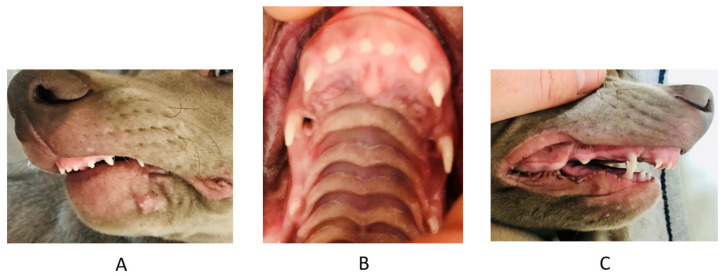
Third case. Extraoral and intraoral photographs at deciduous dentition stage. (**A**) left side, (**B**) palate, (**C**) right side.

**Figure 9:**
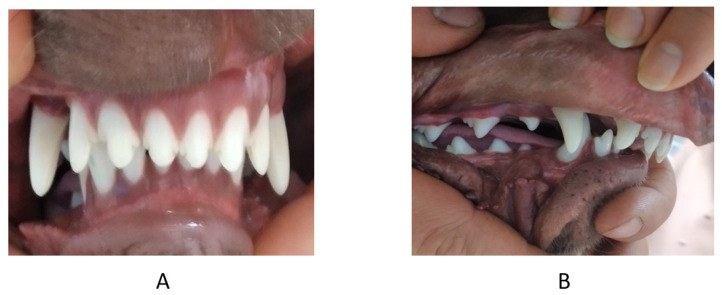
Third case. Intraoral photograph in full occlusion before the treatment. Visible asymmetry of arches with a disturbed plane. (**A**) en face, (**B**) right side.

The last of the patients was initially diagnosed by the breeder who, due to the incorrect size of the mandible at the stage of deciduous dentition, excluded the dog from breeding. He informed the future owner about his observation and recommended a veterinary and dental consultation (Figure 8). The owner presented his dog (six months old) for diagnosis. Distoclusion was found as well as persistent deciduous dentition. The dog was from a regular litter, born on time, without any complications. The mandible is short and symmetrical. Inside, there is an oral recess in the palate: 5 mm deep, intense reddening, no exudate from the cavity, and the mandibular canine is tilted into the space behind the maxillary canine, causing the mandible to deviate to the opposite side. Plane disturbance and severe distoclusion were observed (Figure 9). The owner decided to start orthodontic treatment.

The procedure described above was used, with a proprietary modification of the tooth cap with an elastic rubber part (modelled on a Stockfisch Kinetor) to stimulate the masseter muscles and to induce functional mandibular extension.

**Figure 10:**
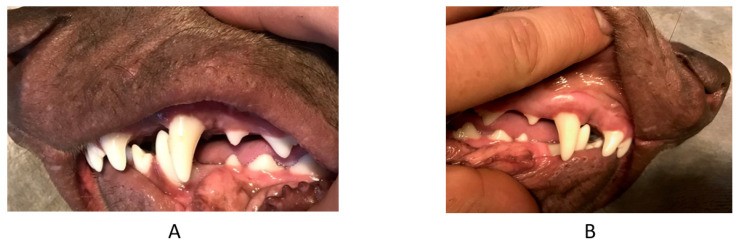
Third case. Intraoral profile photographs 2 months after the start of treatment. (**A**) left side, (**B**) right side.

The patient wore the device for 2 weeks. Then, the appliance was lined, and the rubber element was changed (positioned more to the front). After another two weeks, the orthodontic appliance was removed from the tooth surface with the orthodontic ring forceps. The cement remnants from the teeth were easily removed with an applicator. The mandibular canines after this stage were not sufficiently tilted in the vestibular direction. The owner refused to complete the treatment due to the dog’s behavioral difficulties resulting from adolescence and personal circumstances. Impressions and photographs were taken for a second time 2 months after the end of the treatment (Figure 10).

## 4. Results

German Shepherd. A control visit took place one year after the end of treatment. A permanent mandible extension was obtained with an improvement in the relationship of the canines. The wounds and the deformations of the palate completely disappeared. The arrangement of the teeth in the mandible became symmetrical. Currently, the dog’s profile matches the breed. The mandible developed significantly during the year. The dog’s jaw clamp is strong. The dog was trained as a guard dog throughout the course of treatment. He carried out all the orders with which he was commanded. The owner did not notice any changes in the dog’s behavior, such as reluctance, apathy, and irritation, despite the treatment. The device, due to the fact that it was round, forced the dog to close its jaws on the breakers. The same dog learned to catch with the non-frontal segment, eliminating the risk of possible knocking out of the incisors. For the owner it was very important information due to the future role of the dog as a guard dog.

German Pointer. A control visit took place one year after the end of treatment. A permanent mandible extension was obtained with an improvement in the relationship of the canines. The wound and the palate deformation had completely receded, and no lump or thinning of the mucosa was felt. Currently, the dog’s profile matches the breed. The mandible expanded during the year; the occlusal plane was symmetrical. There was no improvement in the structure of the frontal bone, and asymmetry was still noticed.

**Figure 11:**
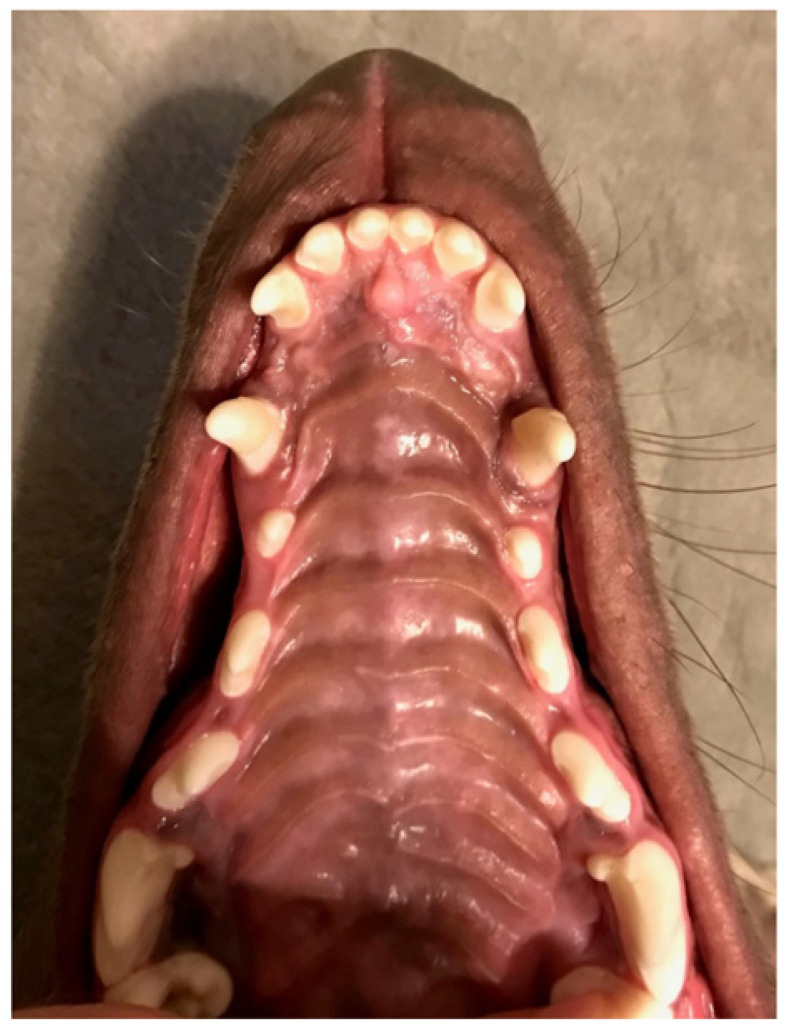
Third case. Permanent teeth in the maxilla 4 months after the start of the treatment. No trace of traumatic occlusion.

Short-haired Weimaraner. The owner returned after two more months. Despite the recommendation of further treatment due to the relation between the maxilla and the mandible and the vertical position of the canines, another treatment was refused due to the uncertain prognosis due to the age and the fact that the dog was excluded from breeding. A characteristic feature of this breed is a scissor bite with vertical teeth position, the effects obtained after the first stage of treatment significantly improved the profile of the mouth and the position of permanent teeth (Figure 11), the owner accepted the termination of the treatment. The dog is under constant observation.

## 5. Discussion

The most common cause of permanent mandibular canine tilting is a persistent deciduous tooth or a tooth removed from the oral cavity too late. A characteristic feature of persistent teeth is a long root, which has not been resorbed or has been only partially resorbed, while there is an erupting or erupted permanent tooth nearby [4,5]. Another cause of canine tilting is distoclusion, and it can also become aggravated by functional inhibition of mandibular growth or a malformation. The most common complication of an incorrectly positioned mandibular fourth permanent tooth is a perforation of the palate and the appearance of an oronasal fistula.

Depending on the age of the animal, several treatments can be used.

General dental methods are tooth extraction or selective grinding. Due to the location and the size of the canine, tooth extraction should be performed under general anesthesia, which may be detrimental to the animal. Extracting a tooth should be done carefully in order not to break the tooth, damage the alveolar process, or break the mandible. An additional disadvantage of this method is that it can later contribute to further pathologies, such as a hanging tongue, improper hold of objects or food, and deepening the asymmetry of the occlusal plane. The second method is selective tooth grinding, i.e., cutting the tooth or softening it. This procedure does not eliminate possible mandibular deviation, and it may require endodontic treatment of the tooth [3,4,8,9,10].

Other methods are therapeutic and orthodontic treatment.

**Figure 12:**
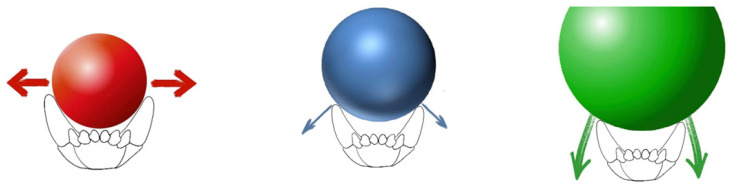
Selecting the size of the ball to the lower interdental width. The red ball only stretches and thus protrudes the teeth. The blue one is intrusive and tilts the teeth. The green ball is only intrusive.

Therapeutic methods include myotherapy, for example, playing with a ball. The most important thing is to correctly match the ball to the interdental width in the mandibular arch (Figure 12). The prerequisite for proper therapeutic treatment is systematic exercise with the dog and multiple, correct bites of the ball [8].

Orthodontic treatment requires specialized and individual appliances. Orthodontic treatment can be used if the teeth are of an appropriate height. The treatment was most often undertaken on seven-month-old puppies in the available literature.

One of the most effective methods of orthodontic treatment is functional treatment. The appliance works due to the forces generated by the muscles pressing the mandible against the maxilla.

Success with a low construction bite depends on stimulation to contract, the strength of the muscles, and the frequency of jaw movements. An example of such an appliance is an inclined plane. It works like an occlusal splint: with each press, it slowly moves the teeth along the slopes of the plane and, affecting on the condylar processes, moving the mandible downwards and forwards. Thus, it affects both the teeth and the joints. As with the activators used successfully in humans, the mandible moves forward as a consequence of the growth of the mandibular condyle, backwards and upwards.

The phylogenetic and ontogenetic properties of the articular cartilage make it possible to influence the growth in the condylar process and therefore remodel the joint. In humans, this mechanism is caused by stretching the lateral pterygoid muscle belly, particularly its upper insertion (Petrovicz) [2].

In treatment with a functional appliance, it is important to investigate the cause and align the center lines on the models, which is difficult in the oral cavity of an animal due to the length of the body of the mandible. Therefore, analysis of the temporomandibular joint should be carried out in great detail. The position of the mandible in relation to the maxilla without occlusion in the lateral sections, i.e., the rest position, determines how much the patient can be lifted in a construction bite. The next examined element is the jaw closing path. Lateral, sagittal deviations or rotation should be noted. Rotation is rarely seen due to the structure of the dog’s temporomandibular joints, i.e., the hinge joint. Premature contact should be examined, especially if the mandibular canines are hidden behind the maxillary canines. When planning and designing the orthodontic appliance, the inclination of the planes can be adjusted to correct the asymmetry. Limiting the abnormality to a disturbance only on one side allows adjusting the planes so that one side is active and the other is passive, i.e., balancing.

**Figure 13:**
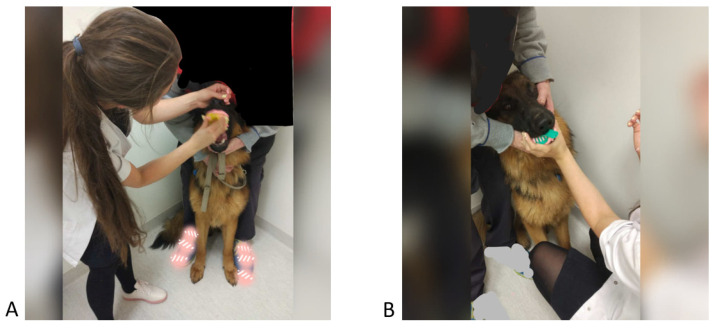
Taking impressions of the maxillary (**A**) and mandibular (**B**) arches of the patient.

The appliance can be made with different materials, such as metal [10] or plastic. Due to the properties of metals, their hardness and stiffness, parts of the opposing tooth may break or chip. Acrylic appliances are the most common, mainly due to their low price, plasticity, versatility, and easy adjustment, even in the clinic. However, the authors of the article have determined through experience that it is not advisable to make acrylic appliances directly inside the patient’s mouth due to the fact that it may burn the mucosa or irritate the pulp of the abutment teeth during the polymerization phase [11,12,13,14,15,16,17,18,19,20,21,22,23,24,25,26,27,28,29]. The appropriate distance between the apparatus and the mucosa can be included using the indirect method in a technical laboratory. This allows maintenance of the correct physiology, the natural flow of saliva in all spaces, and removal of excess cement if it flows into cavities or removal of excess food. The indirect method requires taking impressions to prepare plaster models on which the technician will work. Taking impressions on two upper trays, selected according to the width of the animal’s mouth, allows mapping of the base. In the mandibular arch, it additionally allows the tongue to be moved away; it creates space for the operator’s fingers to be positioned and also allows holding of the mandible while the impression is taken. (Figure 13). The quality of the impression depends not only on the skill of the person taking the impression but also on the good cooperation between the owner and the animal. By reason of the price and the speed of setting, making impressions with alginate mass is recommended. The accuracy of the models speaks in favor of silicone mass impressions, but it also requires a longer time of keeping the impression tray in the patient’s mouth. It is also more difficult to remove hair or whiskers from the silicone mass without an unpleasant experience for the animal.

When designing and constructing the appliance, it should be noted that the safe thickness of the acrylic, which prevents it from cracking during use, is 2 mm [1,2]. Smoothing also prevents abrasions or cuts, which cannot be achieved by making an apparatus inside the mouth. It is recommended to make the appliances from a transparent and colorless material, which will allow the owner to take proper care of hygiene between visits to the clinic [12,15].

**Figure 14:**
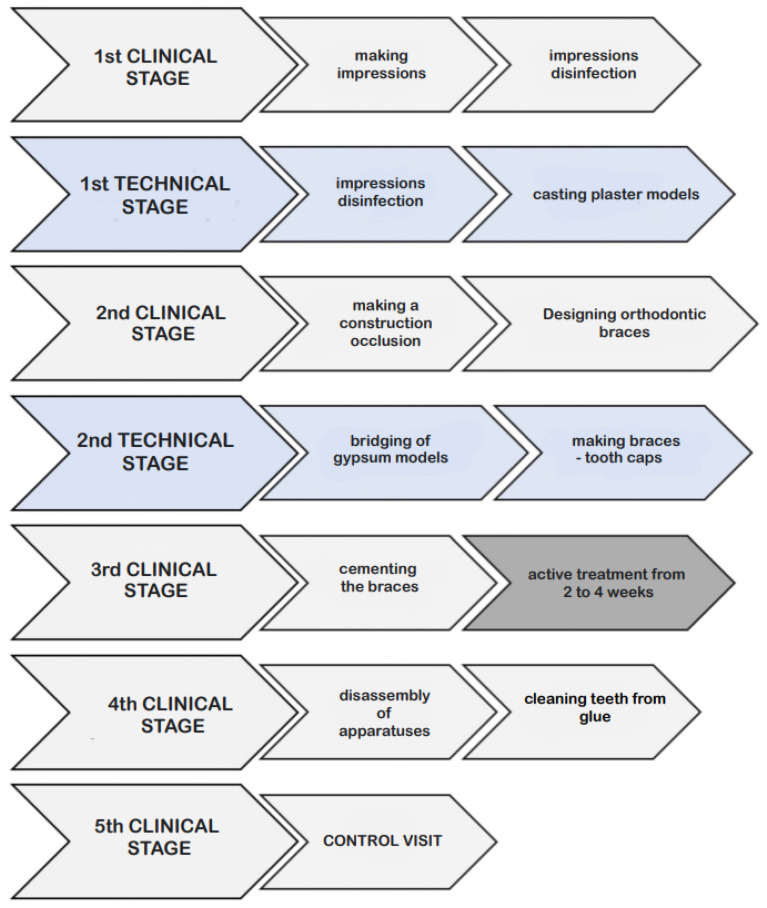
Protocol of the procedure.

Acrylic does not have adhesive properties; therefore, binding materials such as cement are used. The use of glass ionomer cement removes the requirement of etching the enamel with orthophosphoric acid [12,13,14,29]. Acid etching of enamel causes micro-damage in the microscopic image; therefore, this type of material is no longer used when cementing temporary pieces [1]. Glass ionomer cement releases fluoride during the use of orthodontic implants [1]. The high adhesive strength and the lack of postoperative hypersensitivity ensure optimal results when placing orthodontic implants (Figure 14).

**Figure 15:**
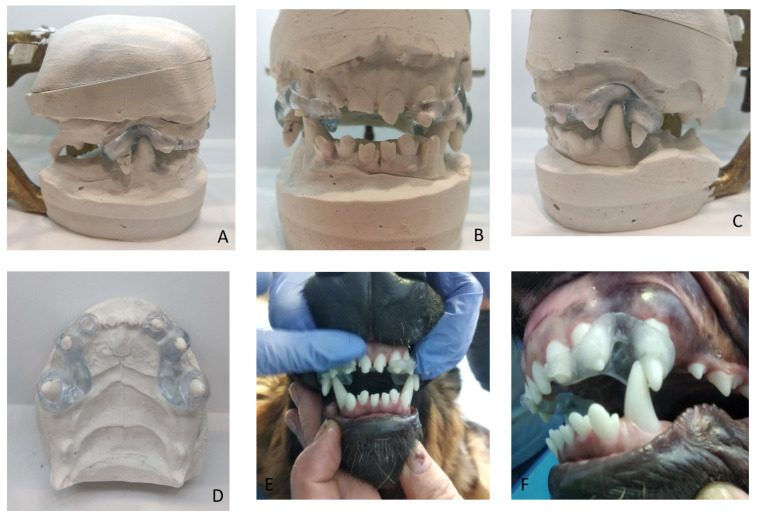
The cap appliance. On models (**A**–**D**) and after cementing (**E**,**F**).

In the first two cases, dental caps were modified, and their elements were adjusted to the anatomy of the dogs’ teeth. Two planes were chosen so that there was no tooth displacement or excessive tilting of the mandible on the passive side. There was constant pressure on the tooth that was supposed to protrude on the active side, according to a strictly defined vector, i.e., forward and sideways. The plane near the maxillary canine was vertical and on the side of the flews, at an inclination consistent with the biological capacity of the mandibular tooth and taking into account the bone thickness around the root (Figure 15). Both cases are examples of an economical appliance that can tilt a tooth quickly and without loosening it, both in humans and in animals. According to the literature and the authors’ observations, the active time of wearing the appliance is approximately a month. This appliance is an alternative to correction with fixed orthodontic braces, without damaging the teeth or periodontal tissues [15].

**Figure 16:**
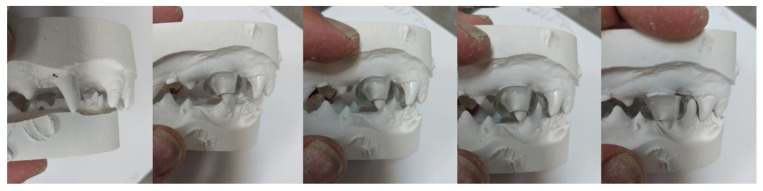
The sequence of the forward movement of the lower jaw and the canine guidance.

**Figure 17:**
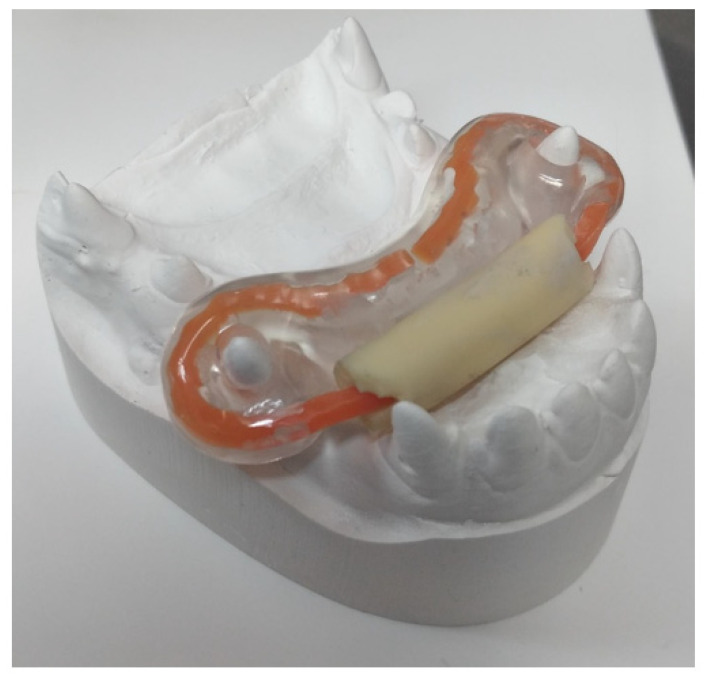
Modification of the activator with a rubber tube.

In the third case, an adjusted plate of a spring appliance was used, mainly based on the top plate of the Stockfisch appliance with its active tongue loops, on which a rubber hose was stretched and secured against slipping. This provoked the desire to bite forwards (Figure 16) and increased muscle tension (Figure 17). The owner noticed that the flews were tenser after treatment in comparison to the state before the treatment.

## 6. Conclusions

The development of medicine allows more treatment options for our pets who, like us, have become victims of the conveniences of the 21st century. The low awareness of pet owners has allowed the reproduction of individuals with skull deformities, such as French Bulldogs. Therapeutic treatment allows for the prevention of many abnormalities. The cases described in this article prove that early orthodontic treatment of linguoverted mandibular canines can be successful in dogs. The treatment is short, and the protocol described above allows the procedure to be performed without a need for sedation, which is important in the case of a developing young organism, and it reduces costs for owners or veterinary clinics. Orthodontic treatment is an excellent alternative to selective grinding of permanent teeth or extraction treatment—the most radical option. Regardless of the cynological reservations referring to the treatment of malocclusion in purebred and show dogs, a too narrow inter-canine gap should be subjected to medical intervention in the interest of the animal.

## Data Availability

The data analyzed for the study are available from the corresponding author upon reasonable request.
